# Integral structured Co–Mn composite oxides grown on interconnected Ni foam for catalytic toluene oxidation

**DOI:** 10.1039/c8ra10102g

**Published:** 2019-02-25

**Authors:** Xueding Jiang, Weicheng Xu, Shufeng Lai, Xin Chen

**Affiliations:** School of Environment and Chemical Engineering, Foshan University Foshan 528000 China jgreatding@163.com xwc1030@163.com

## Abstract

Considering the three-dimensional ordered network of Ni foam-supported catalysts and the toxicity effects of volatile organic compounds (VOCs), the design of proper active materials for the highly efficient elimination of VOCs is of vital importance in the environmental field. In this study, a series of Co–Mn composite oxides with different Co/Mn molar ratios grown on interconnected Ni foam are prepared as monolithic catalysts for total toluene oxidation, in which Co_1.5_Mn_1.5_O_4_ with a molar ratio of 1 : 1 achieves the highest catalytic activity with complete toluene oxidation at 270 °C. The Co–Mn monolithic catalysts are characterized by XRD, SEM, TEM, H_2_-TPR and XPS. It is observed that a moderate ratio of Mn/Co plays significant effects on the textural properties and catalytic activities. From the XPS and H_2_-TPR characterization results, the obtained Co_1.5_Mn_1.5_O_4_ (Co/Mn = 1/1) favors the excellent low-temperature reducibility, high concentration of surface Mn^3+^ and Co^3+^ species, and rich surface oxygen vacancies, resulting in superior oxidation performance due to the formation of a solid solution between the Co and Mn species. It is deduced that the existence of the synergistic effect between Co and Mn species results in a redox reaction: Co^3+^–Mn^3+^ ↔ Co^2+^–Mn^4+^, and enhances the catalytic activity for total toluene oxidation.

## Abstract 2

A series of Co–Mn oxides with different Co/Mn molar ratios grown on interconnected Ni foam were prepared as monolithic catalysts for total toluene oxidation.

## Graphical abstract

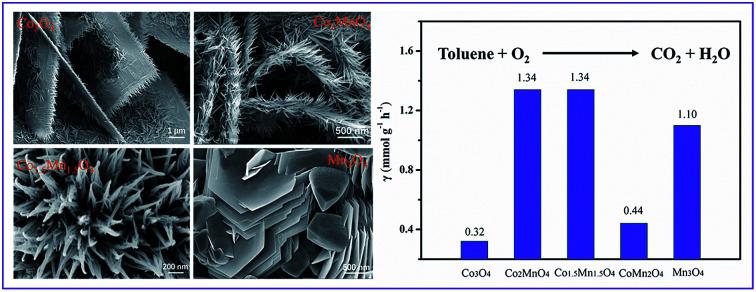

## 1. Introduction

With increasing energy consumption and emission of air pollutants from industrial processes and transport vehicles, the large amounts of volatile organic compounds (VOCs) in the atmosphere are harmful to human health and the living environment.^1–4^ Catalytic oxidation is one of the low-cost and most promising technologies for VOC degradation in recent decades.^5–8^ In addition, the commercial catalysts are designed by dispersing the active components into honeycomb ceramics (2MgO·2Al_2_O_3_·5SiO_2_). Unfortunately, this method always causes inhomogeneous active components in impregnation processes, which results in lower catalytic efficiency and limited application.^9^ Meanwhile, some studies have been performed on the development of metallic substrates or modular catalysts in the environmental field.^9–15^ Among the metallic substrates, Ni foam has special advantages such as a low cost, high porosity, rich accessible electroactive sites, high thermal conductivity, and mass transfer ability,^13,15,16^ so may be considered as a better alternative to honeycomb ceramics.

Various transition metal oxides as active components in reaction processes have been extensively studied to replace noble metals over the past several years, because they have low cost, unique structural morphology, adequate catalytic activity and high thermal stability.^3,17–21^ Co_3_O_4_, a transition metal oxide, has been proven to excellent catalytic activity in numerous reactions duo to its superior physical–chemical properties.^22–27^ Additionally, extensive efforts have revealed that the synergistic effect of Co species and other transition metal oxides has dramatically enhanced the redox properties and catalytic activities due to the formation of a solid solution, compared with single oxides.^11,28–31^ For example, Jiang *et al.*^30^ reported the preparation of Mn–Co–O_*x*_ nanocubes with different Mn/Co molar ratios derived from metal–organic frameworks. It is shown that hierarchical Mn–Co–O_*x*_ mixed metal oxides exhibited the better redox properties, more exposed active sites and superior oxidation performances of total toluene oxidation. Chen *et al.*^11^ synthesized mesoporous CoMnAl mixed metal oxides from layered double hydroxide (LDH) for total benzene oxidation. Results showed that CoMn_2_AlO-550 displayed rich oxygen vacancies and optimal catalytic activity due to the formation of a solid solution of cobalt–manganese oxides. Therefore, one effective strategy on introducing Mn species into Co_3_O_4_ phase to develop multi-element mixed oxides, can be successfully used for removal of VOCs and improved redox properties.

Herein, a series of binary Co–Mn oxides with different Co/Mn molar ratios embedded in interconnected Ni foam were well prepared *via* a simple hydrothermal reaction, and their catalytic performances were investigated in the total toluene oxidation (a model reaction). Furthermore, integral structured Co–Mn composite oxides grown on interconnected Ni foam were characterized by numerous techniques, such as XRD, SEM, TEM and XPS, to understand the correlation between physical–chemical properties and reactivity of Co–Mn composite oxides. This study is to provide guidelines for the rational fabrication of integral structured Co-based composite oxides grown on interconnected Ni foam for effectively catalytic toluene oxidation.

## 2. Experimental

### 2.1 Preparation of catalysts

An aqueous salt solution (with Co/Mn molar ratios of 3/0, 2/1, 1/1, 1/2 and 0/3, Co^2+^ + Mn^2+^ = 3 mmol) was prepared by dissolving Co(NO_3_)_2_·6H_2_O and Mn(NO_3_)_2_ into 40 mL of deionized water. The 12 mmol of solid urea was then added to the homogeneous salt solution. The cleaned Ni foam (4 cm × 6 cm) was immersed in the precursor solution, and were then transferred into in a 50.0 mL Teflon-lined autoclave at 95 °C for 12 h in an electric oven. After cooling down to indoor temperature, the covered Ni foams with array precursors were washed several times with deionized water and ethanol. Finally, the covered Ni foams were heated at 400 °C in air for 2 h to form the composite oxide phase. These calcined Ni foams were designated as Co_3−*x*_Mn_*x*_O_4_ (*x* = 0, 1, 1.5, 2 and 3), respectively.

### 2.2 Catalyst characterization

The crystal structure of as-prepared structured catalysts was characterized by X-ray diffraction (XRD) (Japan, D/max 2500) with Cu-Kα radiation (2*θ* = 5–90°) at 40 kV and 30 mA.

The pore size distribution, pore volume and the Brunauer–Emmett–Teller (BET) surface areas of as-prepared structured catalysts were measured by using a Micromeritics ASAP2020 at −196 °C. Before the tests, all structured catalysts were degassed at 120 °C for 2.5 h.

The surface morphology and microstructure of as-prepared structured catalysts were carried out by using scanning electron microscopy (SEM, SU-8020) and transmission electron microscope (TEM, JEOL 2100F), respectively.

The H_2_ temperature programmed reduction (H_2_-TPR) measurements were carried out on an Automated Catalyst Characterization System (Autochem 2920, MICROMERITICS). Prior to H_2_-TPR, the structured catalyst (1 cm × 3 cm) were heated under a gas flow of 5% O_2_/He (25 mL min^−1^) from indoor temperature to 300 °C. After cooling to room temperature, the structured catalyst was reduced under a gas flow of 10% H_2_/Ar (30 mL min^−1^) with at a heating rate of 10 °C min^−1^.

X-ray photoelectron spectroscopy (XPS) measurements were recorded by using an XLESCALAB 250Xi electron spectrometer from VG Scientific with monochromatic Al Kα (1486.6 eV) radiation, and the peak positions were calibrated by the C 1s peaks at 284.6 eV.

### 2.3 Catalytic performance test

The catalytic activities of total toluene oxidation over the Co_3−*x*_Mn_*x*_O_4_ composite oxides were performed in a continuous-flow quartz tube (i.d. = 10 mm) using about 0.24 g covered Ni foams (2 cm × 4 cm), the covered Ni foams were bent into the reaction tube. The test was carried out in the temperature range of 180–300 °C under 1000 ppm toluene balanced with air at a total flow rate of 120 mL min^−1^. Thus, a weight hourly space velocity (WHSV) of 30 000 mL g^−1^ h^−1^ or a gas hour space velocity (GHSV) of 12 000 h^−1^ was applied for the whole experiment. The toluene conversion was persistently measured, and was reached typically at the final temperature for 1 h in each testing temperature. The concentrations of toluene and products (CO or CO_2_) in the effluent gas was analyzed by using an on-line GC-2014 with two flame ionization detector (FID). The catalytic activities over the Co_3−*x*_Mn_*x*_O_4_ composite oxides were calculated as following equation:1
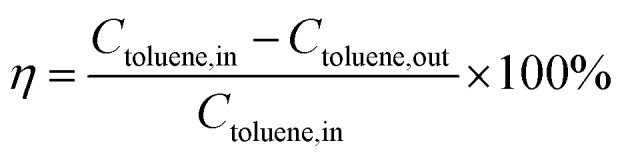
where *η*, *C*_toluene,in_ and *C*_toluene,out_ are the total VOCs conversion, toluene in the inlet and outlet gas, respectively.

The apparent activation energy (*E*_a_) values of were calculated by the equations:2
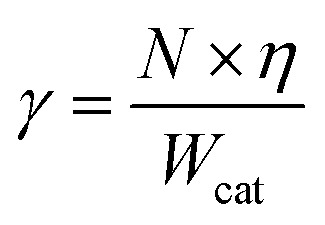
3
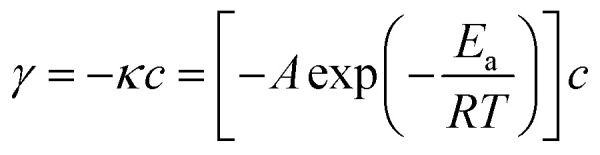


## 3. Results and discussion

**Fig. 1:**
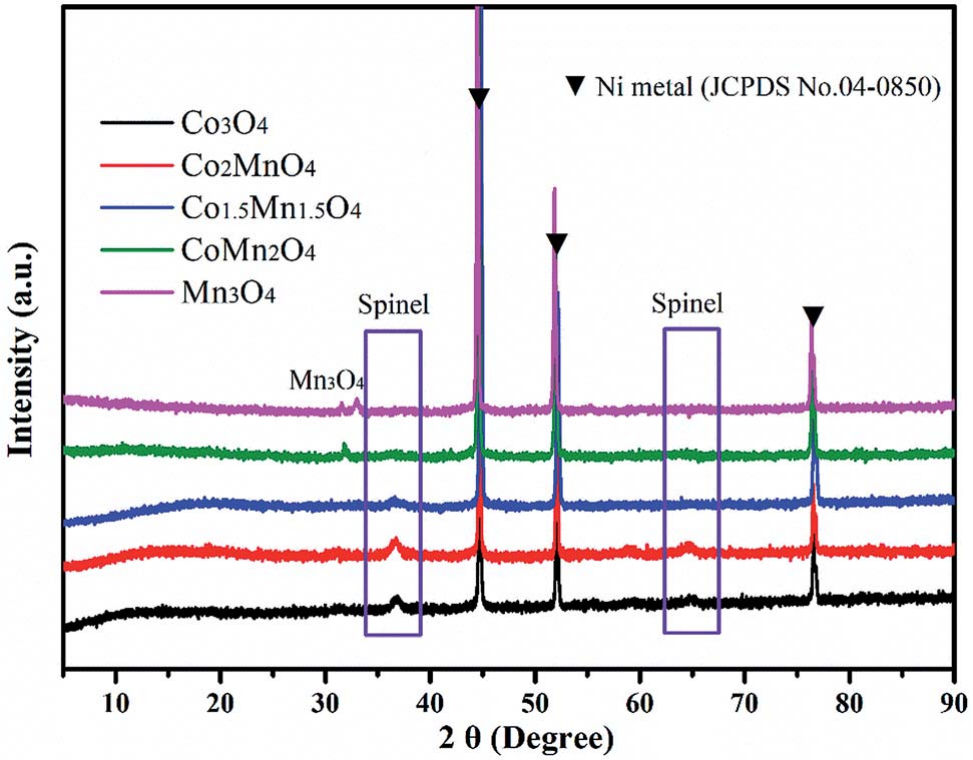
The XRD patterns of as-synthesized Co–Mn composite oxides grown on interconnected Ni foam.

As shown in XRD patterns of as-synthesized Co–Mn composite oxides, the strong peaks at 2*θ* = 44.8°, 51.5° and 76.3° could be well indexed to nickel metal (JCPDS no. 04-0850). For Co-rich catalysts (Co_3_O_4_, Co_2_MnO_4_ and Co_1.5_Mn_1.5_O_4_), the diffraction peaks at 36.3° and 64.8° can be attributed to the (311) and (440) planes of the spinel oxide phase (Co_3_O_4_, JCPDS 43-1003), respectively.^32^ For Mn-rich catalysts (CoMn_2_O_4_ and Mn_3_O_4_), the characteristic peaks at around 30.9° and 32.4° were corresponds to Mn_3_O_4_ phase (JCPDS no. 89-4837). According to the XRD results, it could be found that the Co_1.5_Mn_1.5_O_4_ catalyst with the molar ratio of Co/Mn = 1/1 has the lower intensity of the diffraction peak, inferring that there is a low crystallinity which will result in abundant crystal defects (Fig. 1).

**Fig. 2:**
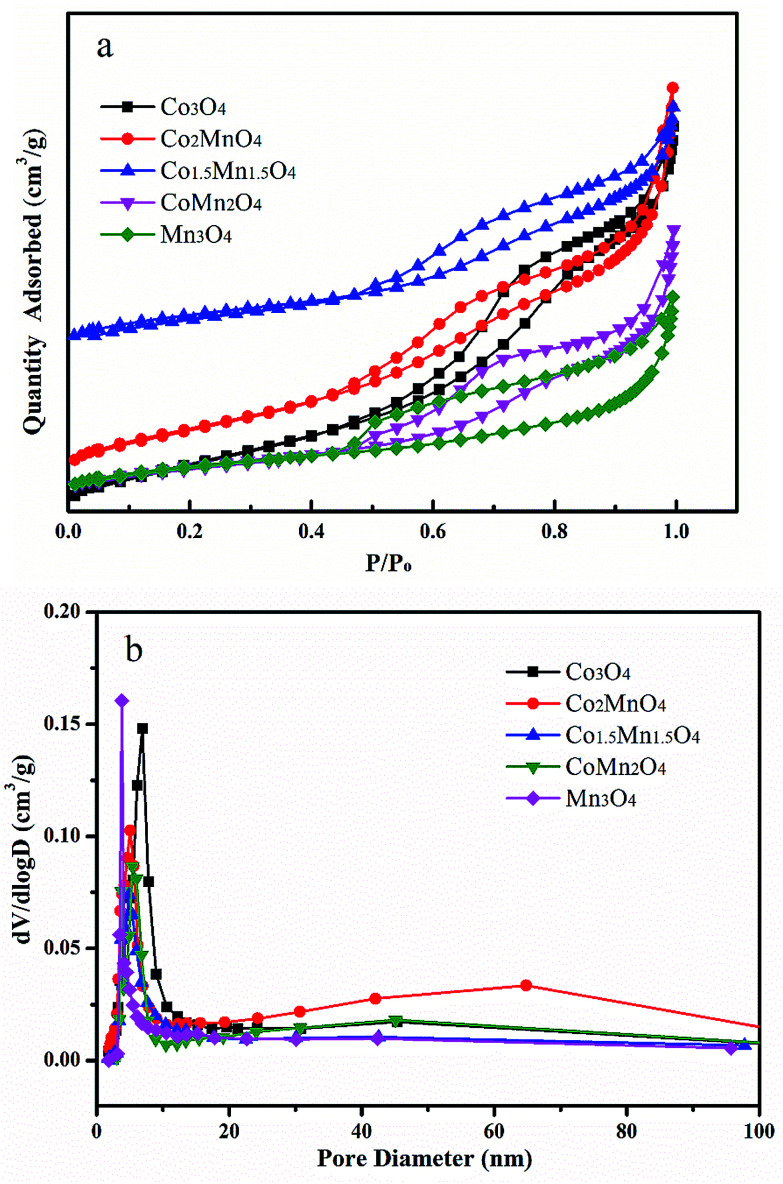
(a) Nitrogen adsorption/desorption isotherms and (b) pore size distributions of as-prepared Co–Mn composite oxides grown on interconnected Ni foam.

The nitrogen adsorption/desorption isotherms and pore size distributions of as-prepared Co–Mn composite oxides grown on interconnected Ni foam are tested to further research the porous structure, as shown in Fig. 2. With the increased Mn species doped into Co_3_O_4_, the specific surface area and pore volume gradually decreases, as summarized in Table 1. There is a type IV sorption isotherms with a type H2 hysteresis loop for each of the samples (Fig. 2a), indicating the presence of homogeneous mesopores. The average pore sizes of as-prepared Co–Mn samples keep in a narrow size distribution (6.8–8.8 nm), as described in Fig. 2b.

**Table 1:** BET data and catalytic activity of as-prepared Co–Mn composite oxides

Samples	BET surfaces area (m^2^ g^−1^)	Pore diameter (nm)	Total pore volume (cm^3^ g^−1^)	*T*_10_ °C	*T*_50_ °C	*T*_90_ °C	*γ* a (mmol g^−1^ h^−1^)
Co_3_O_4_	26.22	0.056	7.8	255	273	277	0.32
Co_2_MnO_4_	25.76	0.061	8.1	245	264	268	1.34
Co_1.5_Mn_1.5_O_4_	14.20	0.033	8.8	240	263	267	1.34
CoMn_2_O_4_	13.99	0.036	8.5	250	275	287	0.44
Mn_3_O_4_	13.95	0.029	6.8	240	255	280	1.10

a The specific toluene reaction rates over all the catalysts were calculated at 270 °C.

**Fig. 3:**
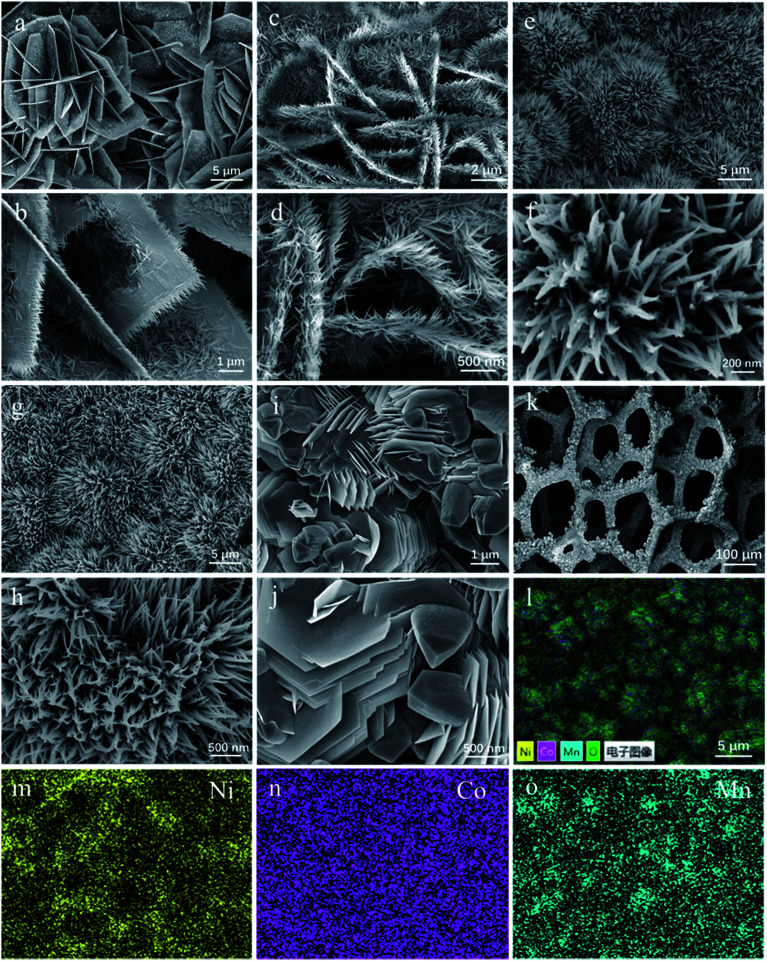
The SEM images of (a and b) Co_3_O_4_, (c and d) Co_2_MnO_4_, (e and f) Co_1.5_Mn_1.5_O_4_, (g and h) CoMn_2_O_4_, (i and j) Mn_3_O_4_ samples and (k) covered Ni foam; (l–o) elemental mapping images of Ni, Co and Mn.

After hydrothermal reactions, the surface of Ni foams is orderly covered *via* uniform coverage of vertical Co/Mn oxide arrays, as demonstrated in Fig. 3k. The morphologies of as-prepared Co–Mn arrays in Ni foam have changed with the increased Mn species doping into Co_3_O_4_ arrays. The Co_3_O_4_ sample mainly exhibits a series of intertwined and hexagonal nanosheets with the epitaxial nanowires in a parallel fashion, as shown in Fig. 3a and b. When the atomic ratio of Co : Mn is about 2 : 1, the nanowires grow significantly at the edge of the nanosheets in Fig. 3c and d. Compared with the Co_3_O_4_ sample, the SEM images (Fig. 3e–h) on the Co_1.5_Mn_1.5_O_4_ and CoMn_2_O_4_ samples have obvious changes, showing that a large number of nanowires are self-assembled into urchin shapes with a diameter of 5–10 μm. For Mn_3_O_4_ sample in Fig. 3i and j, it could be observed that many hexagonal nanosheets with a diameter of 1–2 μm aggregated on the margins of sample, and the morphology of Mn_3_O_4_ sample is not completely uniform. In addition, EDX mapping (Fig. 3l–o) shows that Co, Ni, Mn elements are uniformly distributed on the surface of Ni foam.

**Fig. 4:**
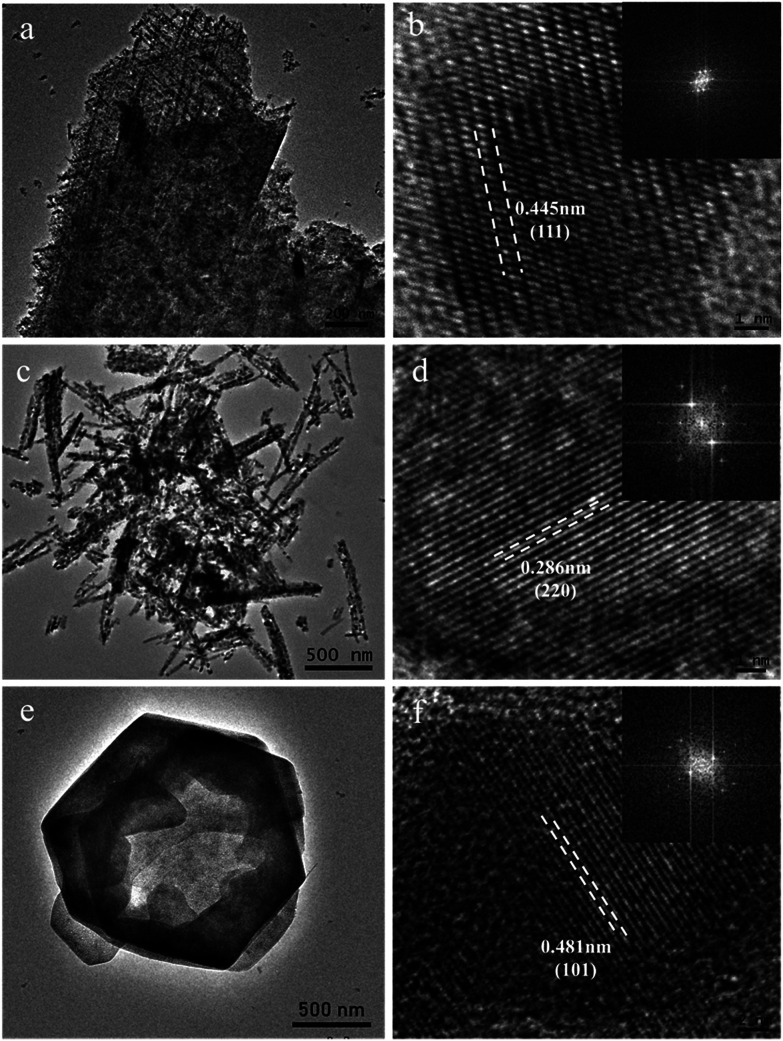
TEM and HRTEM images of (a and b) Co_3_O_4_, (c and d) Co_1.5_Mn_1.5_O_4_ and (e and f) Mn_3_O_4_ samples (inset is the fast-Fourier-transform pattern).

The micro-structure of as-prepared Co–Mn composite oxides was investigated *via* TEM analysis. As shown in Fig. 4a and b, a series of nanowires are uniformly distributed on the extremely rough surface of nanosheets with tiny pores inside, which is consistent with SEM results. A HRTEM image of Co_3_O_4_ is shown in Fig. 4b, the high-resolution lattice fringes calculated to be 0.445 nm could be indexed to the (111) lattice plane of Co_3_O_4_ phase. TEM image in Fig. 4c further reveals that the morphology of Co_1.5_Mn_1.5_O_4_ sample is composed of a large number of nanowires with a diameter of 50–70 nm, the corresponding HRTEM image in Fig. 4d shows that the lattice fringes with an interplanar spacing of 0.286 nm is assigned to the (220) lattice plane of Co_3_O_4_ phase. No lattice streaks of manganese oxides are observed, indicating the formation of a solid solution between Co and Mn species. The TEM image of Mn_3_O_4_ sample in Fig. 4e exhibits hexagonal nanosheets with a diameter of 2 μm, some lattice fringes belong to the (101) lattice planes of manganese oxides (lattice fringes = 0.48 nm) can be observed. The formation of a solid solution is favorable for low temperature reduction, electron transfer, abundant surface oxygen vacancy and oxidation–reduction reaction, which will be further confirmed *via* other characterization analysis.

**Fig. 5:**
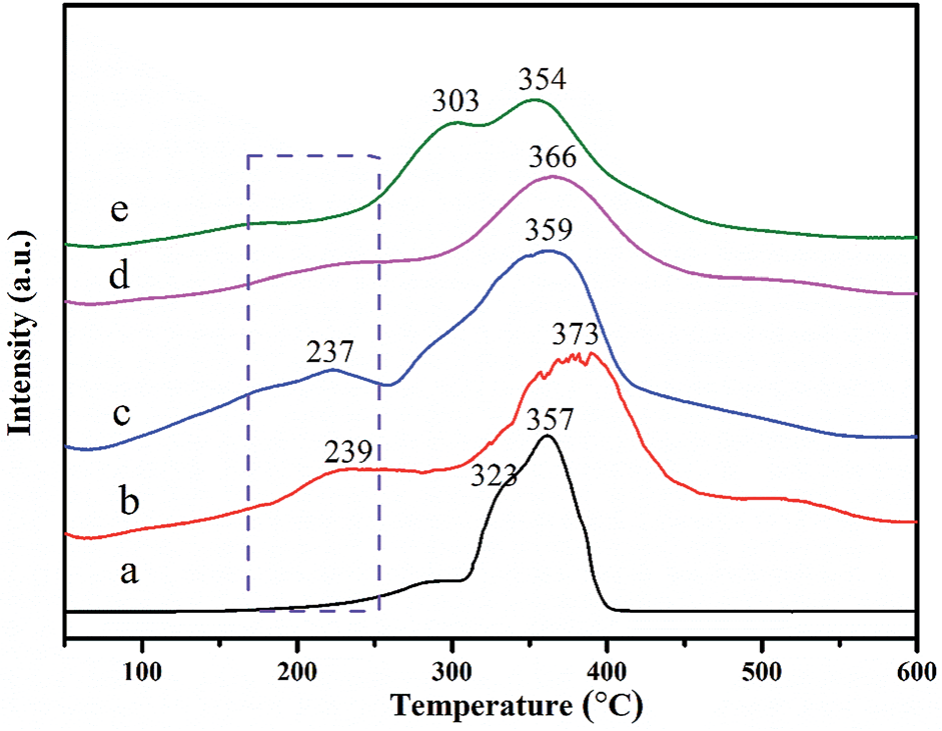
H_2_-TPR curves of as-prepared Co–Mn composite oxides grown on interconnected Ni foam, labels (a)–(e) correspond to the Co_3_O_4_, Co_2_MnO_4_, Co_1.5_Mn_1.5_O_4_, CoMn_2_O_4_ and Mn_3_O_4_, respectively.

The reducibility of as-prepared Co–Mn composite oxides grown on interconnected Ni foam was carried out by the H_2_-TPR test. The H_2_-TPR curves of Co–Mn composite oxides are showed in Fig. 5, there are mainly two to three reduction peaks over the Co–Mn catalysts. The reduction step of Co_3_O_4_ phase is Co^3+^ → Co^2+^ → Co^0^, and the reduction step of manganese oxides is Mn^4+^ → Mn^3+^ → Mn^2+^.^2,33^ For Co_3_O_4_ sample with three reduction peaks, the one peak in the low temperature range of 200–300 °C is associated with the reduction of surface Co^3+^ into Co^2+^, the overlapping peaks in the low temperature range of 300–400 °C is attributed to the further reduction of bulk Co^3+^ into Co^2+^ and metallic cobalt.^19,34^ The TPR profiles of Mn_3_O_4_ sample exhibits two separated reduction peaks at 303 °C and 354 °C, which are assigned to the following two reduction processes: Mn^4+^ → Mn^3+^ and Mn^3+^ → Mn^2+^, respectively. In addition, compared to single Co_3_O_4_ and Mn_3_O_4_ catalysts, the first reduction peaks of Co–Mn mixed phase catalysts are gradually shifted to low temperature regions, suggesting that their low temperature reducibility is improved *via* the synergistic effect of Co and Mn species. All the results reveal that the Co_1.5_Mn_1.5_O_4_ is easier to be reduced in the low temperature range of 200–300 °C than other monolithic array catalysts, meaning a facilitated redox process and a better catalytic performance.

**Fig. 6:**
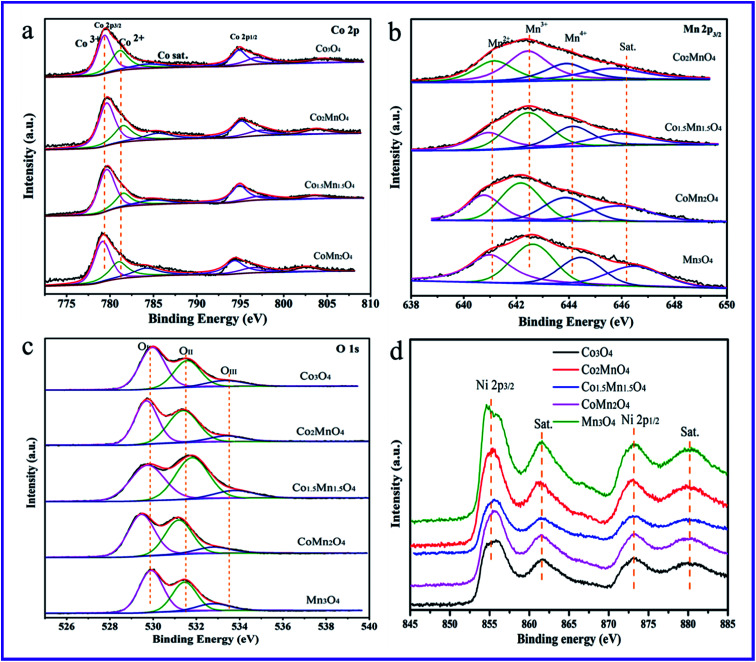
High-resolution XPS spectra of (a) Co 2p, (b) Mn 2p_3/2_, (c) O 1s and (d) Ni 2p over the as-prepared Co–Mn composite oxides.

Fig. 6 shows the Co 2p, Mn 2p_3/2_, O 1s and Ni 2p XPS spectra of the five samples. And the related results of chemical states are summarized in Table 2. The Ni 2p XPS spectra in Fig. 6d shows four peaks at the binding energy of 856.4, 861.5, 873.1 and 880.2 eV attributed to Ni 2p_3/2_, satellite peak of Ni 2p_3/2_, Ni 2p_1/2_ and the satellite peak of Ni 2p_1/2_, respectively.^35,36^ The Ni 2p XPS spectra primarily come from Ni foams. As for Co 2p XPS spectra (Fig. 6a), the peaks at the binding energy of 779.9, 781.5 and 785.5 eV are related to surface Co^3+^, Co^2+^ and satellite peak of Co species, respectively.^17,18,31^ In addition, the Mn 2p_3/2_ XPS spectrum in Fig. 6b is deconvoluted into four peaks. According to the H_2_-TPR results, it could be observed that there is the presence of Mn^4+^ and Mn^3+^ species. Thus, the three peaks of Mn 2p_3/2_ XPS spectrum at the binding energy of 641.2, 642.5, 644.3 and 646.1 eV are attributed to the surface Mn^2+^, Mn^3+^, Mn^4+^ and the satellite to the surface Mn^3+^ species, respectively.^4^ According to the XPS results, the surface molar ratio of Co^3+^/Co^2+^ follows the sequence of Co_1.5_Mn_1.5_O_4_ (2.54) > Co_2_MnO_4_ (1.96) > CoMn_2_O_4_ (1.65) > Co_3_O_4_ (1.43), suggesting that the Co^3+^/Co^2+^ molar ratio of the Co_1.5_Mn_1.5_O_4_ is highest than those of other Co–Mn composite oxides. Similarly, the surface molar ratio of Mn^3+^/Mn_total_ follows the sequence of Co_1.5_Mn_1.5_O_4_ (0.471) > CoMn_2_O_4_ (0.456) > Co_2_MnO_4_ (0.413) > Mn_3_O_4_ (0.38). Surface oxygen vacancies would be generated to maintain electrostatic balance once a lower manganese state (Mn^3+^) existed in the catalysts according to the following process:^37^–Mn^4+^–O^2−^–Mn^4+^– → –Mn^4+^–□–Mn^3+^– + 1/2O_2_□ represents an oxygen vacancy in the catalysts.

**Table 2:** Surface chemical compositions of as-prepared Co–Mn composite oxides

Samples	Co^3+^	Co^2+^	Co^3+^/Co^2+^	Mn^4+^	Mn^3+^	Mn^2+^	O_I_	O_II_	O_III_
Co_3_O_4_	0.588	0.412	1.43	—	—	—	0.547	0.347	0.106
Co_2_MnO_4_	0.663	0.337	1.96	0.293	0.413	0.294	0.471	0.428	0.155
Co_1.5_Mn_1.5_O_4_	0.717	0.283	2.54	0.276	0.471	0.253	0.435	0.464	0.101
CoMn_2_O_4_	0.622	0.378	1.65	0.262	0.456	0.282	0.504	0.390	0.106
Mn_3_O_4_	—	—	—	0.263	0.368	0.367	0.535	0.355	0.11

The higher ratio of Mn^3+^/Mn_total_ implies the presence of rich oxygen vacancies. Moreover, there is the electronic transfer between Co and Mn species to provide a redox reaction: Co^3+^–Mn^3+^ ↔ Co^2+^–Mn^4+^.^11^

Fig. 6c shows that the O 1s XPS spectra are mainly deconvoluted into three kinds of surface oxygen species. The peaks located at the binding energy of 529.8, 531.2 and 533.2 eV could be attributed to characteristic of the surface lattice oxygen (O_I_, O^2−^), adsorbed oxygen (O_II_, O_2_^−^, O_2_^2−^ or O^−^) and adsorbed oxygen-containing hydrocarbons (O_III_, OH, H_2_O) species, respectively.^38,39^ As shown in Table 1, the ratio of surface lattice oxygen (O_I_) remarkably decreases with introducing Mn species into Co_3_O_4_ phase, thereinto, the Co_1.5_Mn_1.5_O_4_ exhibits the lowest surface lattice oxygen concentration. It is to be noted that the electrophilic surface adsorbed oxygen species (O_ads_ = O_II_ + O_III_) play vital roles in the deep VOCs oxidation, the formation of surface adsorbed oxygen species is due to the presence of surface oxygen vacancies (V_O_). According to the Co 2p and Mn 2p results, surface oxygen vacancies are beneficial for maintaining electrostatic balance and participating in the redox reaction. Therefore, it is reasonable that the Co_1.5_Mn_1.5_O_4_ will exhibit an excellent catalytic activity for total toluene oxidation.

**Fig. 7:**
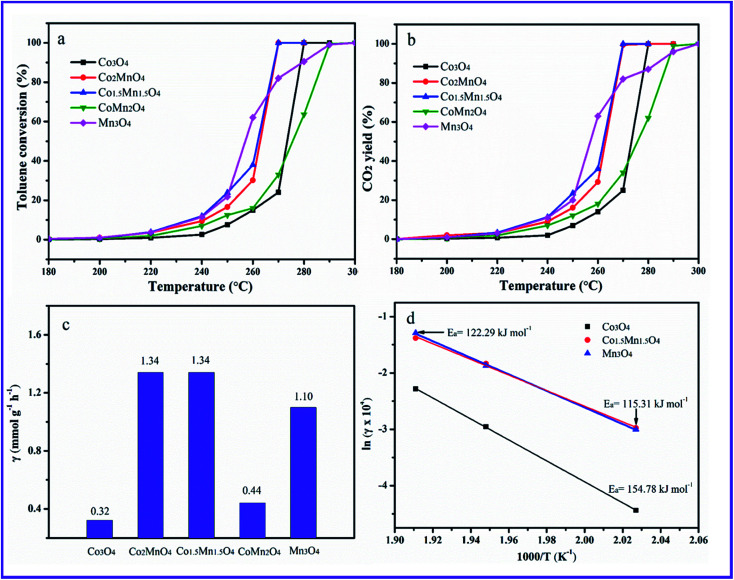
(a) Toluene catalytic activity, (b) CO_2_ yields and (c) reaction rate at 270 °C of the as-prepared catalysts; (d) Arrhenius plots for total toluene oxidation over the Co_3_O_4_, Co_1.5_Mn_1.5_O_4_ and Mn_3_O_4_ catalysts. Reaction conditions: *C*_toluene_ = 1000 ppm at WHSV = 30 000 mL h^−1^ g^−1^.

The toluene catalytic activity of as-prepared Co–Mn composite oxides as a function of temperature is shown in Fig. 7A. It could be observed that the catalytic combustion of toluene over catalysts increases with the increased temperature. As presented, the Mn species doping into Co_3_O_4_ arrays has a significant influence on the catalytic activity of toluene oxidation. The optimal molar ratio of Co/Mn resulted in the large increase of catalytic activity for toluene oxidation. The reaction temperatures *T*_10_, *T*_50_ and *T*_90_ at which the toluene conversions of 10%, 50% and 90% is converted to CO_2_, are summarized in Table 1, which are used to compare the catalytic activities for toluene oxidation. For Co_3_O_4_ sample, *T*_10_, *T*_50_ and *T*_90_ values are 255, 273 and 277 °C, respectively, and toluene could be completely converted into CO_2_ and H_2_O at about 280 °C. The *T*_10_ an *T*_50_ of Mn_3_O_4_ sample are superior to those of Co_3_O_4_ sample, whose 10% and 50% toluene conversions could be obtained at 240 and 255 °C, whereas the *T*_90_ value over Mn_3_O_4_ obviously shifts to high temperature region. It can be seen that adding manganese to cobalt improved the toluene conversions. According to the temperature values of complete toluene oxidation (*T*_99_), the catalytic activity over the five samples follows the sequence of Co_1.5_Mn_1.5_O_4_ (270 °C) ≈ Co_2_MnO_4_ (270 °C) > Co_3_O_4_ (280 °C) > CoMn_2_O_4_ (290 °C) > Mn_3_O_4_ (300 °C), suggesting the Co_1.5_Mn_1.5_O_4_ exhibits the highest catalytic activity for total toluene oxidation. CO_2_ concentration is also detected during catalytic toluene reaction, and CO_2_ yield is shown in Fig. 7b. It can be concluded that toluene is completely converted into CO_2_. The specific toluene reaction rates are calculated at 270 °C, as shown in Fig. 7c. The Co_2_MnO_4_ and Co_1.5_Mn_1.5_O_4_ catalysts exhibits higher reaction rate with 1.34 mmol g^−1^ h^−1^, which is four point two times the reaction rate of Co_3_O_4_ catalyst with 0.32 mmol g^−1^ h^−1^. Furthermore, the activation energies (*E*_a_) over the catalysts are calculated by the Arrhenius plots in Fig. 7d. The *E*_a_ decreases from 154.78 to 115.31 kJ mol^−1^ in the sequence of Co_3_O_4_, Mn_3_O_4_ and Co_1.5_Mn_1.5_O_4_, which is related to the catalytic performance for toluene oxidation.

**Fig. 8:**
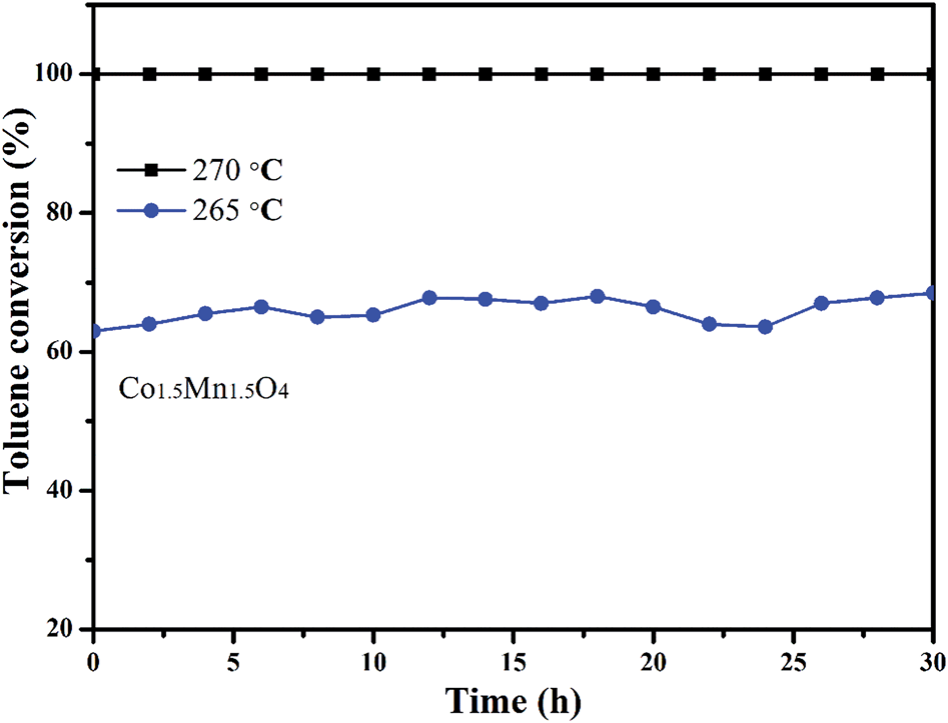
Stability test of Co_1.5_Mn_1.5_O_4_ catalyst at different temperatures.

In addition, the stability of Co_1.5_Mn_1.5_O_4_ with the best catalytic activity is also tested by a long-term time at different temperatures, as shown in Fig. 8. Under the complete removal temperature (270 °C), the toluene conversions with approximately 100% value are nearly unchangeable after 30 h test. When the reaction temperature is decreased to 265 °C, the toluene conversion apparently decreased from 100% to 64%, there are five percent fluctuation on toluene conversion. The activity increased slightly after 30 h reaction at 265 °C, which is due to the adsorption of water molecules on the active sites in begin and the exothermic reaction of toluene combustion. This result indicates that Co_1.5_Mn_1.5_O_4_ catalyst own outstanding catalytic activity and sustainability.

Previous studies revealed that the reactivity of a catalyst for VOCs oxidation can be greatly associated with physical–chemical properties including the specific surface area, surface active species, oxygen vacancies, reducibility and synergistic effect of metal cation redox couple. The oxides with a formula of AB_2_O_4_ (A_3_O_4_ when A = B), which is recognized as a spinel phase.^27^ In the Co_3_O_4_ structure, the Co^2+^ cations occupy tetrahedral holes to form a tetrahedral CoO_4_ sites and the Co^3+^ cations occupy octahedral holes to obtain octahedral CoO_6_ sites. According to the literature,^40^ the crystal structure of MnCo_2_O_4_ displays the higher valence Mn and Co cations in the (110) plane, and the Mn cations can substitute the octahedral site and octahedral site of Co cations. The high valence Mn cations occupy partial inactive Co^2+^ sites, which promotes the reduction and synergistic effect of cobalt and Mn species. In this work, introducing Mn species into Co_3_O_4_ construction varied catalytic performances and physical–chemical properties. Although the specific surface area and pore volume gradually decreases with the increased Mn species into Co_3_O_4_, the Co-rich catalysts (Co_2_MnO_4_ and Co_1.5_Mn_1.5_O_4_) exhibits superior catalytic performances for total toluene oxidation, indicating no direct relation between the specific surface area and the reactivity. According to the H_2_-TPR results, the Co_1.5_Mn_1.5_O_4_ owns low-temperature reducibility than other monolithic array catalysts due to the facilitated redox process from the synergistic effect of Co and Mn species. The above H_2_-TPR results are confirmed by XPS. There is the electronic transfer between Co and Mn species to provide a redox reaction: Co^3+^–Mn^3+^ ↔ Co^2+^–Mn^4+^. In addition, abundant surface oxygen vacancies are beneficial for maintaining electrostatic balance and participating in the redox reaction. These results indicates the insignificant effect of low-temperature reducibility, the synergistic effect of Co and Mn species and surface oxygen vacancies on the catalytic activity for total toluene oxidation.

## 4. Conclusions

A series of unique Co–Mn oxides with different Co/Mn molar ratios and morphology grown on interconnected Ni foam were well prepared *via* an ordinary hydrothermal reaction, in which Co_1.5_Mn_1.5_O_4_ with the molar ratios of 1 : 1 displayed the highest catalytic activity for total toluene oxidation. It is observed that the Co/Mn molar ratio played significant influence on the textural properties and catalytic activities of obtained catalysts. Pure Co_3_O_4_ sample mainly exhibited a series of intertwined and hexagonal nanosheets with the epitaxial nanowires, pure Mn_3_O_4_ sample showed many hexagonal nanosheets with a diameter of 1–2 μm, the Co–Mn composite oxides mainly appeared urchin shapes self-assembled *via* a large number of nanowires. Based on the temperature values of complete toluene oxidation, the activity of toluene catalytic oxidation follows Co_1.5_Mn_1.5_O_4_ ≈ Co_2_MnO_4_ > Co_3_O_4_ > CoMn_2_O_4_ > Mn_3_O_4_. From the characterization results of XPS and H_2_-TPR, introducing Mn element into Co_3_O_4_ sample resulted in the formation of a solid solution between Co and Mn species, improved the low-temperature reducibility, concentration of surface Mn^3+^ and Co^3+^ species, and surface oxygen vacancies. It is deduced that he synergistic effect of Co and Mn species provided a redox reaction: Co^3+^–Mn^3+^ ↔ Co^2+^–Mn^4+^ and enhanced the catalytic activity for total toluene oxidation.

## Conflicts of interest

There are no conflicts to declare.

## Supplementary Material
